# Knockdown of Splicing Complex Protein PCBP2 Reduces Extravillous Trophoblast Differentiation Through Transcript Switching

**DOI:** 10.3389/fcell.2021.671806

**Published:** 2021-05-20

**Authors:** Danai Georgiadou, Souad Boussata, Remco Keijser, Dianta A. M. Janssen, Gijs B. Afink, Marie van Dijk

**Affiliations:** Reproductive Biology Laboratory, Amsterdam Reproduction and Development Research Institute, Amsterdam UMC, University of Amsterdam, Amsterdam, Netherlands

**Keywords:** differentiation, invasion, splicing, placenta, extravillous trophoblast, HELLP syndrome, PCBP2

## Abstract

Mutations in the *LINC-HELLP* non-coding RNA (*HELLPAR*) have been associated with familial forms of the pregnancy-specific HELLP syndrome. These mutations negatively affect extravillous trophoblast (EVT) differentiation from a proliferative to an invasive state and disturb the binding of RNA splicing complex proteins PCBP1, PCBP2, and YBX1 to *LINC-HELLP*. In this study, by using both *in vitro* and *ex vivo* experiments, we investigate if these proteins are involved in the regulation of EVT invasion during placentation. Additionally, we study if this regulation is due to alternative mRNA splicing. HTR-8/SVneo extravillous trophoblasts and human first trimester placental explants were used to investigate the effect of siRNA-mediated downregulation of *PCBP1*, *PCBP2*, and *YBX1* genes on the differentiation of EVTs. Transwell invasion assays and proliferation assays indicated that upon knockdown of PCBP2 and, to a lesser extent, YBX1 and PCBP1, EVTs fail to differentiate toward an invasive phenotype. The same pattern was observed in placental explants where PCBP2 knockdown led to approximately 80% reduction in the number of explants showing any EVT outgrowth. Of the ones that still did show EVT outgrowth, the percentage of proliferating EVTs was significantly higher compared to explants transfected with non-targeting control siRNAs. To further investigate this effect of PCBP2 silencing on EVTs, we performed whole transcriptome sequencing (RNA-seq) on HTR-8/SVneo cells after PCBP2 knockdown. PCBP2 knockdown was found to have minimal effect on mRNA expression levels. In contrast, PCBP2 silencing led to a switch in splicing for a large number of genes with predominant functions in cellular assembly and organization, cellular function and maintenance, and cellular growth and proliferation and the cell cycle. EVTs, upon differentiation, alter their function to be able to invade the decidua of the mother by changing their cellular assembly and their proliferative activity by exiting the cell cycle. PCBP2 appears to be a paramount regulator of these differentiation mechanisms, where its disturbed binding to *LINC-HELLP* could contribute to dysfunctional placental development as seen in the HELLP syndrome.

## Introduction

The HELLP syndrome is a pregnancy complication occurring in approximately 0.5% of pregnancies and is characterized by Hemolysis, Elevated Liver enzymes, and Low Platelets in the mother. Symptoms specific to the HELLP syndrome can arise as a severe manifestation of preeclampsia, but they can also occur without *de novo* hypertension, which is an essential criterion for the diagnosis of preeclampsia. The HELLP symptoms can occur from the 20th week of pregnancy or even postpartum, but their cause originates in the first trimester. In those first months reduced invasion of extravillous trophoblasts (EVT) into the maternal decidua leads to hampered spiral artery remodeling causing insufficient blood flow to the fetus (Abildgaard and Heimdal, 2013). The dysfunction of the EVT can be due to a failure to undergo Epithelial Mesenchymal Transition (EMT) in which the trophoblasts transform from a proliferative epithelial trophoblast into an invasive mesenchymal type of cell (Davies et al., 2016). How this failure is triggered is still unknown.

Previously, by genome-wide linkage analysis of Dutch women with a familial form of the HELLP syndrome, we identified a long non-coding RNA on chromosome 12q23, *LINC-HELLP* (official gene symbol *HELLPAR*) (Van Dijk et al., 2012). It was shown that mutations in *LINC-HELLP* identified in HELLP families negatively affected EVT differentiation either by inducing proliferation rate or by causing cell cycle exit as shown by a decrease in both proliferation and invasion (van Dijk et al., 2015). Furthermore, as LincRNAs predominantly function through interactions with proteins, multiple interacting proteins were identified predominantly clustering in two functional networks, i.e., RNA splicing and the ribosome. The RNA splicing proteins further investigated were YBX1, PCBP1, and PCBP2. Binding of *LINC-HELLP* RNA to the YBX1 and PCBP2 proteins was confirmed to be influenced by the HELLP mutations carried (van Dijk et al., 2015).

YBX1 is one of three Y box binding protein family members containing a highly conserved cold shock protein domain and is able to bind to both DNA and RNA. It thereby has multiple functions such as regulating apoptosis, cell proliferation, differentiation, and stress response through regulation of transcription and translation, mRNA splicing, DNA repair, and mRNA packaging (Suresh et al., 2018).

PCBP1 and PCBP2 are two of the four widely expressed poly(C)-binding protein family members. PCBP1 is an intronless highly similar homolog of PCBP2, and both are abundantly expressed in different tissues and species. PCBP1 and PCBP2 each contain three RNA binding domains with high affinity for binding to C-rich polypyrimidine motifs (Makeyev and Liebhaber, 2002). Similar to YBX1, they also have been implicated in regulating cell proliferation and differentiation, as such behaving as oncogenes (Guo and Jia, 2018).

In the current study, we further investigated the role of the RNA splicing proteins YBX1, PCBP1, and PCBP2 in the regulation of EVT invasion during first trimester placenta development. For this we first performed siRNA mediated knockdown on EVT-like HTR-8/SVneo cells and first trimester placental explants followed by experiments measuring proliferation and invasion. The data show that specifically knockdown of PCBP2 leads to EVT differentiation failure. Next, we studied if this regulation of EVT invasion by PCBP2 is associated with alternative mRNA splicing, which is a prominent function of PCBP2 (Ji et al., 2016). We again performed knockdown experiments in HTR-8/SVneo cells followed by whole genome transcriptome sequencing (RNA-Seq). The data obtained was analyzed to detect both differential expression as well as differential transcript usage, showing that PCBP2 mainly affects splicing and not so much mRNA levels *per se*. Several transcripts of important EMT regulators are affected by PCBP2 providing evidence for the major role of PCBP2 in EVT differentiation in which its disturbed binding to *LINC-HELLP* could contribute to dysfunctional placental development.

## Materials and Methods

### Cell Culture and Transfection

HTR-8/SVneo EVT cells were obtained from ATCC (LGC Standards, France) and cultured in RPMI media supplemented with FBS, HEPES, sodium pyruvate, glucose, and pen/strep at 37 °C, 5% CO_2_. For transfection experiments, in 6 well plates 200,000 HTR-8/SVneo cells in 2 ml medium were transfected with 160 pmol *PCBP1, PCBP2*, or *YBX1* siRNAs (Qiagen, Germany, Flexitube Genesolution, a package of four preselected siRNAs targeting the different genes) or non-targeting siRNAs (Qiagen) using 10 μl XtremeGENE HP transfection reagent (Sigma). Forty-eight hours after transfection, the cells were harvested and stored at −80 °C for RNA or protein preparations or harvested to be used in invasion assays.

### RNA Isolation and Quantitative PCR

RNA was isolated from transfected HTR-8/SVneo cells (described above) using the RNeasy Mini Kit (Qiagen) followed by cDNA synthesis using random hexamers and M-MLV reverse transcriptase (Promega). Quantitative PCRs were performed in triplicate on a Lightcycler 480 instrument using SYBR Green Supermix (Roche) according to the manufacturer’s protocol in combination with primers specific for YBX1, PCBP1, PCBP2, and PSMD4 and YWHAZ as reference genes. For validation of transcript switching primers specific for FLNB exon 30 and reference exons, NCOR2 exon 8 and reference exons, and EXOC7 exon 7 and reference exons were used. Primer sequences can be found in Supplementary Table 1. The relative quantity (RQ) of gene expression was calculated using RQ = 2^–Δ*Ct*^, where ΔCt = Ct target gene − Ct reference genes (geometric mean).

### Western Blot

HTR-8/SVneo cell lysates from transfected HTR-8/SVneo cells (described above) were prepared using RIPA buffer supplemented with cOmplete protease inhibitor cocktail (Roche) and PhosSTOP phosphatase inhibitor cocktail (Roche). Samples were prepared in LDS sample buffer (Life technologies), run on a NuPAGE 4–12% Bis-Tris Gel (Invitrogen), and subsequently blotted on an Immobilon PVDF membrane. Blotted proteins were visualized using Revert total Protein stain (Li-Cor) according to protocol and measured on the Odyssey Imaging System (Li-Cor). For visualization of specific proteins, the blots were blocked with blocking buffer (Li-Cor) and incubated overnight at 4°C with primary antibodies YBX1 (1:200, Santa Cruz Biotechnology, sc-101198), PCBP1 (1:200, Santa Cruz Biotechnology, sc-137249), or PCBP2 (1:3,000, Santa Cruz Biotechnology, sc-101136). Next, Goat Anti-Mouse secondary antibodies (1:15,000, LI-COR, 926-32210) were used for 1 h at room temperature. Final imaging of the membrane was done on the Odyssey Imaging System.

### Invasion and Proliferation Assays

To measure invasive capacity, 50,000 transfected and harvested HTR-8/SVneo cells were seeded in medium without FBS on 100 μl 16× diluted (final concentration approximately 0.6 mg/ml) Matrigel (Corning) coated 8.0 μm pore cell culture inserts. To induce invasion the inserts were placed in wells containing medium with FBS. Invasion took place for 48 h after which the membranes were fixed in 4% PFA and cells attached on the inside of the insert were removed. The membranes were mounted in Vectashield (VectorLabs) with DAPI (50 μM) (ThermoFisher) and coverslipped, after which the cells on the underside of the membrane were counted. To count the cells pictures were taken of nine random fields per membrane using a Leica Fluorescent microscope after which ImageJ was used to quantify the number of cells per picture. To measure proliferation, 48 h after siRNA transfection alamarBlue^TM^ reagent (Invitrogen) was added to the cells according to protocol. Four hours later fluorescence was measured on a Synergy microplate reader.

### First Trimester Placental Explants

Human first trimester placenta specimens (*n* = 12) were obtained from the HIS Mouse Facility of the Amsterdam UMC, location AMC, Amsterdam. All material has been collected from donors at the time of elective terminations of pregnancy from whom a written informed consent for the use of the material for research purposes had been obtained by the Bloemenhove clinic. Small fragments (15–20 mg wet weight) of placental villi from 5 to 12 weeks gestation were dissected from the placenta and placed in 48 well culture plates coated with 3 mg/ml collagen I (R&D systems). Transfection was done 24 h later with 80 pmol *PCBP1, PCBP2*, or *YBX1* siRNAs (Qiagen, Germany, Flexitube Genesolution, a package of four preselected siRNAs targeting the different genes) or non-targeting siRNAs (Qiagen) using 2.5 μl XtremeGENE HP transfection reagent (Sigma) in a total volume of 250 μl. Placental villous explants were cultured for a maximum of 5 days at 5% O_2_ and 37 °C in serum free DMEM/F12 media supplemented with pen/strep. Images of the explants were taken on a Leica microscope daily to monitor changes in outgrowth. At day 4 or 5 explants were fixed in 4% PFA and embedded for immunohistochemistry.

### Immunohistochemistry

Embedded first trimester placental explants were sectioned and subjected to standard immunohistochemistry procedures. Antigen retrieval was performed using microwave pre-treatment in sodium citrate buffer. Blocking was done in 5% BSA, followed by overnight primary antibody incubations in 1% BSA at 4 °C. The following primary antibodies were used: YBX1 (1:200, Santa Cruz Biotechnology, sc-101198), PCBP1 (1:200, Santa Cruz Biotechnology, sc-137249), and PCBP2 (1:200, Santa Cruz Biotechnology, sc-101136) that stain for the respective proteins, HLA-G (1:100, Novus Biologicals, NB500-302) that was used as a marker for EVTs, and phosphoH3(Ser10) (1:200, Sigma, 09–797) that was used as a marker for cell proliferation. Finally, Powervision Poly-HRP secondary antibodies were used followed by DAB staining and counterstaining using haematoxilin. ImageJ was used to count the number of proliferating extravillous trophoblasts.

### RNA-Seq

RNA was isolated from transfected HTR-8/SVneo cells (described above) using the RNeasy Mini Kit (Qiagen). RNA-Seq libraries from 4 non-targeting control and 4 PCBP2 silenced samples all obtained in the same experiment were prepared using the KAPA RNA Hyperprep kit including ribodepletion (Roche). The 8 sample libraries were multiplexed across two sequencing lanes and paired-end (2 bp × 150 bp read length) sequencing was performed on an Illumina HiSeq4000 sequencer. The unmapped paired FASTQ files were processed with Cutadapt (v2.9) (Martin, 2011), and Sickle (v1.33) to remove adaptor sequences and trim low-quality bases. Quality control assessment was performed using FastQC (v0.11.9).

### Gene Expression Analysis

STAR aligner (v2.7.3a) (Dobin et al., 2013) was used to align the reads against to the Human reference genome (GRCh38). The BAM alignments were position-sorted and indexed with Samtools (v1.10) and fragment counts were obtained with FeautureCounts (v2.0.0) (Liao et al., 2014). Reads were counted at the gene level and multi-mapped reads were discarded.

All statistical calculations were performed in R programming language (v3.6.3). Differential expression analysis was performed with R package EdgeR (v3.26.8) (Robinson et al., 2009). Only genes with an expression level greater than 0.2 count per million in at least four samples were included in the analysis. Genes exhibiting differential expression with *p* < 0.05 were included in further downstream analyses.

### Differential Transcript Usage

Kallisto (v0.46.2) (Bray et al., 2016) was used to pseudo-align the paired FASTQ files, using an index based on the ENSEMBL GRCh38 Homo sapiens transcriptome. For statistical testing to identify differential transcript usage the transcript-level counts obtained from Kallisto were imported using tximport (v1.12.3) and analyzed with DRIMSeq (v1.12.0) Bioconductor packages (Soneson et al., 2015; Robinson and Nowicka, 2016) following the protocol described by Love et al. (2018) including the following filters for a transcript: it has a count of at least 10 in at least four samples, it has a relative abundance proportion of at least 0.1 in at least 4 samples, and the total count of the corresponding gene is at least 10 in all eight samples.

### Ingenuity Pathway Analysis and MEME Motif Analysis

For functional enrichment analysis Ingenuity Pathway Analysis (Qiagen) was performed on the gene list obtained through the analysis of differential transcript usage. For the MEME motif analysis we constructed a data file containing all intronic regions (up to 500 bp) directly adjacent to the in- and excluded exons or transcription start sites of the genes in Table 2 and the genes of Table 3 that were found to also be differentially spliced in our study. MEME motif enrichment (v5.2.0) (Bailey et al., 2015) was then used to identify significant (*E* value < 0.05) motifs in our data file. The *E*-value of the motif is based on its log likelihood ratio, width, sites, the background frequencies, and the size of the training set. The final width of the motif identified was determined by increasing the maximum width until a peak in relative entropy (mean bit score) of the motif was obtained.

### Statistics

Statistical analyses were performed using Graphpad Prism version 8 software using unpaired *t*-tests in case of two groups or with one-way or two-way ANOVA followed by a Dunnett’s multiple comparison test when comparing multiple groups. P values ≤ 0.05 were considered significant.

## Results

### In EVT-Like Cells RNA Splicing Complex Proteins Affect Differentiation

**FIGURE 1:**
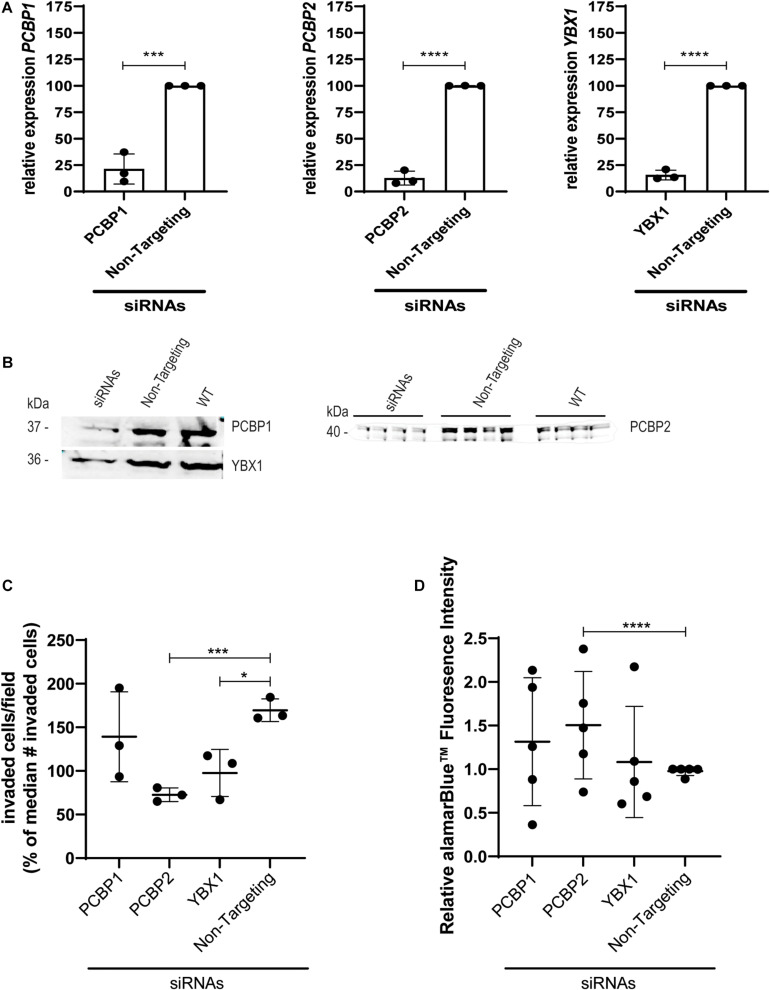
Upon knockdown of YBX1, PCBP1, and PCBP2 EVT-like cells fail to differentiate toward an invasive phenotype. **(A)** qPCR results demonstrating successful mRNA downregulation of *PCBP1*, *PCBP2*, and *YBX1* in HTR-8/SVneo cells after transfection with respective siRNAs. An siRNA not targeting any known mammalian gene was used as a control. Normalization was done using the geometric mean of reference genes *PSMD4* and *YWHAZ*. Experiments were repeated 3 times using 3 replicates per treatment. **(B)** Successful siRNA-induced downregulation of target genes on protein level as shown by Western blot. Equal protein loading control was performed by total protein Revert stain (Supplementary Figure 1). **(C)** Transwell invasion assays show that PCBP2 and YBX1 downregulation leads to a significant decrease in invasive behavior of EVT-like cells. Experiments were repeated 3 times using 6 replicates per treatment. **(D)** Quantification of proliferation using alamarBlue^TM^ solution. The proliferation rate of EVT-like cells with downregulated expression of PCBP2 was increased. Experiments were repeated five times using nine replicates per treatment. Data are presented as mean ± SD and tested with two-way ANOVA followed by Dunnett’s multiple comparisons test or with a Student’s *t*-test in case of two data sets. *Indicates *p* < 0.05; *** indicates *p* < 0.001; **** indicates *p* < 0.0001.

To study the effect of YBX1, PCBP1, and PCBP2 on EVT-like HTR-8/SVneo cell differentiation we transiently transfected the cells with siRNAs directed against the three mRNAs and compared their effect to the non-targeting control siRNAs. Significant knockdown was confirmed on mRNA (Figure 1A) and protein level (Figure 1B) by quantitative PCR and Western blot, respectively. In transwell invasion assays knockdown of YBX1, PCBP1, and PCBP2 led to a significant decrease in the number of invaded cells for siRNAs against YBX1 and PCBP2 (Figure 1C), while knockdown of PCBP1 had no clear effect. On the other hand, knockdown of PCBP2 only significantly increased the proliferation rate of these cells (Figure 1D), while PCBP1 and YBX1 knockdown did not lead to an apparent effect on proliferation. These results together indicate that *in vitro* knockdown of two out of three proteins negatively affect EMT, with PCBP2 providing the strongest effect.

### PCBP2 Knockdown Diminishes Outgrowth of First Trimester Placental Explants

**FIGURE 2:**
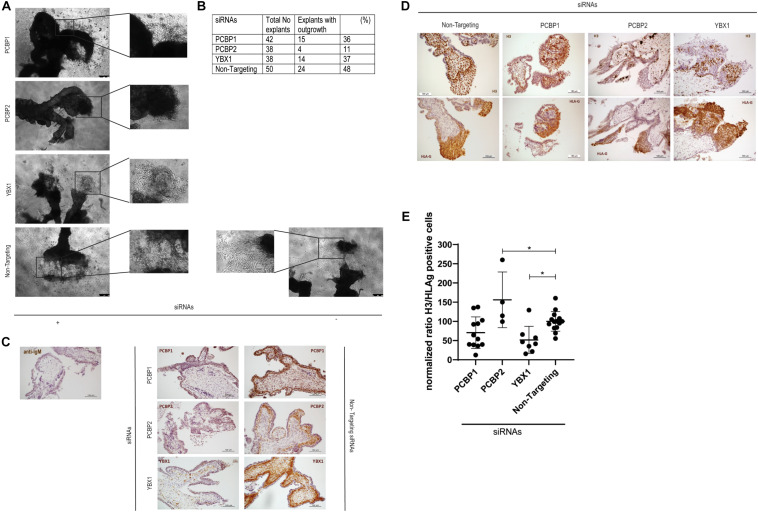
Downregulation of PCBP2 severely affects first trimester placental explant outgrowth. **(A)** Representative images of first trimester placental explants treated with siRNAs targeting *PCPB1*, *PCBP2*, *YBX1*, or non-targeting siRNAs that developed EVT outgrowth. **(B)** Number and percentage of explants that showed EVT outgrowth out of the total number of explants that were treated with the different siRNAs. PCBP2 downregulation leads to the strongest inhibition of explant outgrowth amongst the genes tested. **(C)** Representative images of immunohistochemical staining for PCBP1, PCBP2, and YBX1 proteins in first trimester placental explants transfected with the different siRNAs. The stainings demonstrate successful downregulation of the respective proteins. No staining was observed in mouse IgG control staining. Antibody-specific DAB staining is shown in brown, hematoxylin counterstain was used to stain nuclei blue. **(D)** Representative phosphoH3(Ser10) and HLA-G immunohistochemical stainings of first trimester placental explants after transfection with *PCBP1, PCBP2, YBX1*, or non-targeting siRNAs to identify proliferating cells undergoing mitosis and to indicate the location of EVTs, respectively. **(E)** Quantification of the ratio of H3/HLA-G representing proliferating EVTs indicates a significant induction of proliferating EVTs upon PCBP2 knockdown while YBX1 silencing leads to a reduction of proliferating EVTs. Within the different conditions each dot represents an explant with quantifiable outgrowths. In total this analysis was performed on explants derived from five different first trimester placentas. Data are presented as mean ± SD and tested with one-way ANOVA followed by Dunnett’s multiple comparisons test. *Indicates *p* < 0.05.

To investigate the *ex vivo* effects of knockdown of YBX1, PCBP1, and PCBP2 we transiently transfected human first trimester placental explants. Using non-targeting control siRNAs to transfect the explants leads to almost half of the explants showing EVT outgrowth. Outgrowth is slightly reduced to around 35% when transfecting *PCBP1* or *YBX1* siRNAs, while transfection with *PCBP2* siRNAs led to almost 90% of the explants not demonstrating any EVT outgrowth, which is a reduction compared to non-targeting control siRNAs of around 80% (Figures 2A,B). Knockdown in the explants was confirmed by immunohistochemistry using antibodies recognizing YBX1, PCBP1, and PCBP2 (Figure 2C). Predominant expression of all three proteins was observed in the villous cytotrophoblasts while the syncytiotrophoblast borders of the explants remained negative. Immunohistochemistry was also used to study the amount of proliferating EVTs in the explants that did show EVT outgrowth. EVTs were identified by staining with HLA-G antibody. In parallel sections we also stained for phosphoH3(Ser10) to identify proliferating cells undergoing mitosis (Figure 2D). We quantified the percentage of proliferating EVT and normalized, within different placenta samples, the results obtained in explants treated with *YBX1*, *PCBP1*, or *PCBP2* siRNAs to the results obtained transfecting with non-targeting control siRNAs. This showed that knockdown of PCBP2 led to a significant increase in proliferating EVTs compared to control, while YBX1 siRNAs led to a significant decrease in proliferation (Figure 2E). The combined results obtained *in vitro* and *ex vivo* clearly point out that knockdown of PCBP2 leads to failure of the EVTs to undergo proper EMT.

### PCBP2 Silencing Does Not Have a Pronounced Effect on Differential Gene Expression

**FIGURE 3:**
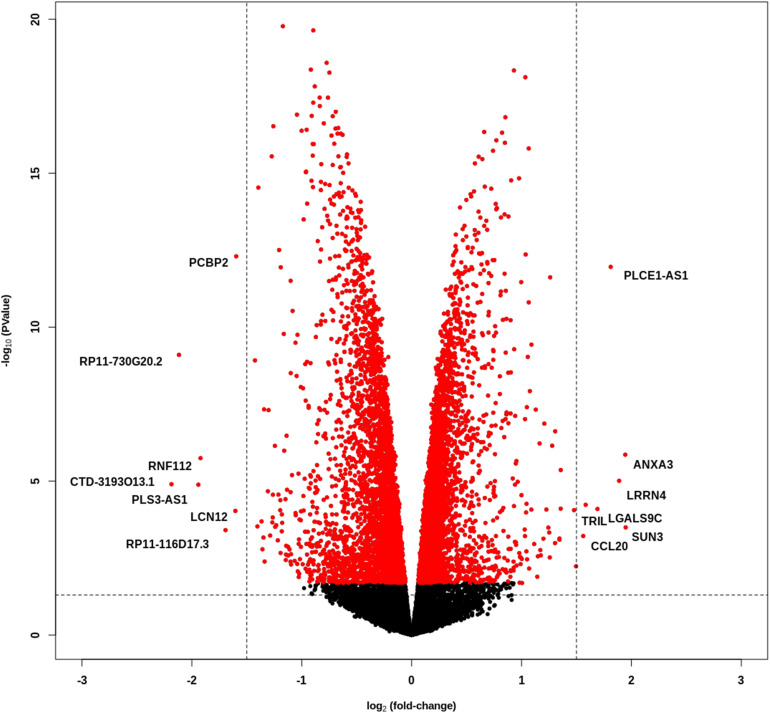
PCBP2 knockdown does not lead to pronounced differential gene expression. Volcano plot of statistical significance against log_2–_fold change between PCBP2 knockdown and non-targeted control samples. The vertical dashed lines indicate a log_2_-fold change < –1.5 and > 1.5, respectively. Red dots denote genes with significant differential expression (*p* < 0.05).

We performed whole transcriptome sequencing (RNA-Seq) on PCBP2 silenced HTR-8/SVneo cells and non-targeted controls, and the obtained data was analyzed. The data show a large number of differentially expressed genes between the control and PCBP2 knockdown samples, albeit at relatively low fold induction (Figure 3). Only 126 genes show more than ± 1 log_2–_fold change in expression levels in combination with an adjusted *p* value < 0.05 (Supplementary Table 2). Of these, only 14 genes have a log_2–_fold change of more than ± 1.5. Additionally, expression levels of these 14 genes are low and only two genes (*PCBP2* and *PLCE1-AS1*) have a logCPM above 0. From these results we concluded that the effect PCBP2 has on EVT differentiation cannot be explained by differential gene expression.

### PCBP2 Silencing Leads to Transcript Switching of Proteins Involved in Cell Differentiation

Because PCBP2 is a known RNA splicing protein, we next sought to analyze differential transcript usage upon PCBP2 silencing. For this, we used DRIMSeq to identify differential splicing between PCBP2 knockdown and control samples. This identified a total number of 310 genes that show differential transcript usage (Supplementary Table 3). These genes were submitted into Ingenuity Pathway Analysis of which the pathway analysis results are presented in Table 1. The top scoring networks and functions show that PCBP2 silencing leads to differential splicing of genes with predominant functions in cellular assembly and organization, cellular function and maintenance, and cellular growth and proliferation and the cell cycle.

**TABLE 1:** Ingenuity Pathway Analysis of differentially spliced genes.

Total # of differentially spliced genes: 310		

Top 5 Molecular and Cellular Functions	− log (*p*-value) range	# Molecules
Cellular Development	2.7–9.1	106
Cellular Growth and Proliferation	2.7–9.1	104
Cell Death and Survival	2.7–9.1	120
Cellular Assembly and Organization	2.7–7.9	95
Cellular Function and Maintenance	2.7–7.9	111

**Top 5 Networks**	**Score**	**# Molecules**

Cancer, Dermatological Diseases and Conditions, Hereditary Disorder	59	31
Cellular Development, Hematological Disease, Hereditary Disorder	46	26
Cellular Assembly and Organization, Cellular Function and Maintenance, Cell Cycle	43	25
Cell Cycle, Cellular Assembly and Organization, Hematological System Development and Function	41	24
Cell-To-Cell Signaling and Interaction, Cell-mediated Immune Response, Cellular Function and Maintenance	34	21

**TABLE 2:** Differentially spliced genes.

Total # of differentially spliced genes: 10 (transcript adjusted *p* value < 0.05; ratio transcript proportion > 1.5 or < 0.67; gene represented by ≥ 2 transcripts; mean counts > 300)							

Gene	Transcript 1	Ratio proportion PCBP2/NT transcript 1	-log(adj p) transcript 1	Transcript 2	Ratio proportion PCBP2/NT transcript 2	-log(adj p) transcript 2	Function
*GNAL*	ENST00000585642.5	21.74	8.3	ENST00000590228.1	0.54	1.5	Stimulatory G protein alpha subunit which couples dopamine type 1 receptors and adenosine A2A receptors.
*IFI16*	ENST00000368131.8	2.14	6.2	ENST00000368132.7	0.56	9.2	Cytokine involved in DNA binding, transcriptional regulation, and protein-protein interactions; Inhibits cell growth in the Ras/Raf signaling pathway.
*CMC1*	ENST00000418849.2	1.82	3.9	ENST00000396610.6	0.56	1.7	Stabilizes the biogenesis of mitochondrial respiratory chain complex IV.
*NCOR2*	ENST00000404121.6	1.78	15.4	ENST00000356219.7	0.60	9.0	Nuclear receptor co-repressor involved in chromatin structure modification
*ANKRD13D*	ENST00000308440.11	1.73	1.8	ENST00000511455.7	0.39	3.0	Interacts with ubiquitin chains for rapid internalization
*FLNB*	ENST00000295956.9	1.57	20.6	ENST00000358537.7	0.46	33.7	Actin-binding protein regulating cytoskeleton dependent cell proliferation and migration; Skipping of exon 30 is associated with EMT
*SMG1P4*	ENST00000381448.8	1.56	2.4	ENST00000380598.4	0.11	2.4	Pseudogene of SMG1; SMG1 is involved in nonsense-mediated mRNA decay as part of the mRNA surveillance complex.
*EXOC7*	ENST00000332065.9	1.54	21.8	ENST00000589210.6	0.66	11.1	Part of exocyst complex which has a critical role in vesicular trafficking and the secretory pathway; Undergoes isoform switching mediated by ESRP1, a splicing factor that regulates EMT
*SEC31A*	ENST00000513858.5	1.53	4.0	ENST00000348405.8	0.53	10.4	Involved in vesicle budding from the endoplasmic reticulum and is required for ER-Golgi transport
*TMEM237*	ENST00000286196.9	1.52	3.0	ENST00000621467.4	0.64	2.3	Involved in WNT signaling; Required for ciliogenesis

**TABLE 3:** Comparison between this study and published results.

Gene	Published skipped exon	Published gene silenced	Our result	Transcript	Ratio proportion PCBP2/NT	−log(adj p)
*RUNX1*	6	PCBP2^#^	Skipped exon 6	ENST00000399240.5	1.55	1.9
*CDK2*	5	PCBP1/2^$^	Skipped exon 5	ENST00000354056.4	1.62	3.6
*SH2B1*	9	PCBP1/2	No exon 9 exclusion			
*TARS2*	14	PCBP1/2; PCBP2 (n.s.)	No exon 14 exclusion			
*CTTN*	11	PCBP1/2; PCBP2	Skipped exon 11	ENST00000376561.7	1.51	5.9
*VKORC1*	2	PCBP1/2	No exon 2 exclusion			
*TRPT1*	7	PCBP1/2	No exon 7 exclusion			
*TFR2*	2	PCBP1/2	Not expressed			
*AP1G2*	3	PCBP1/2	No exon 3 exclusion			
*WNK4*	8	PCBP1/2	Skipped exon 8	ENST00000587705.5	4.42	1.7
*ARHGAP4*	9	PCBP1/2	Not expressed			
*STAT2*	18	PCBP1/2	No exon 18 exclusion			

*^#^Depletion of PCBP2 only; ^$^Co-depletion of PCBP1 and PCBP2; n.s., not significant.*

### Validation of Transcript Switching by Two Distinct Approaches

**FIGURE 4:**
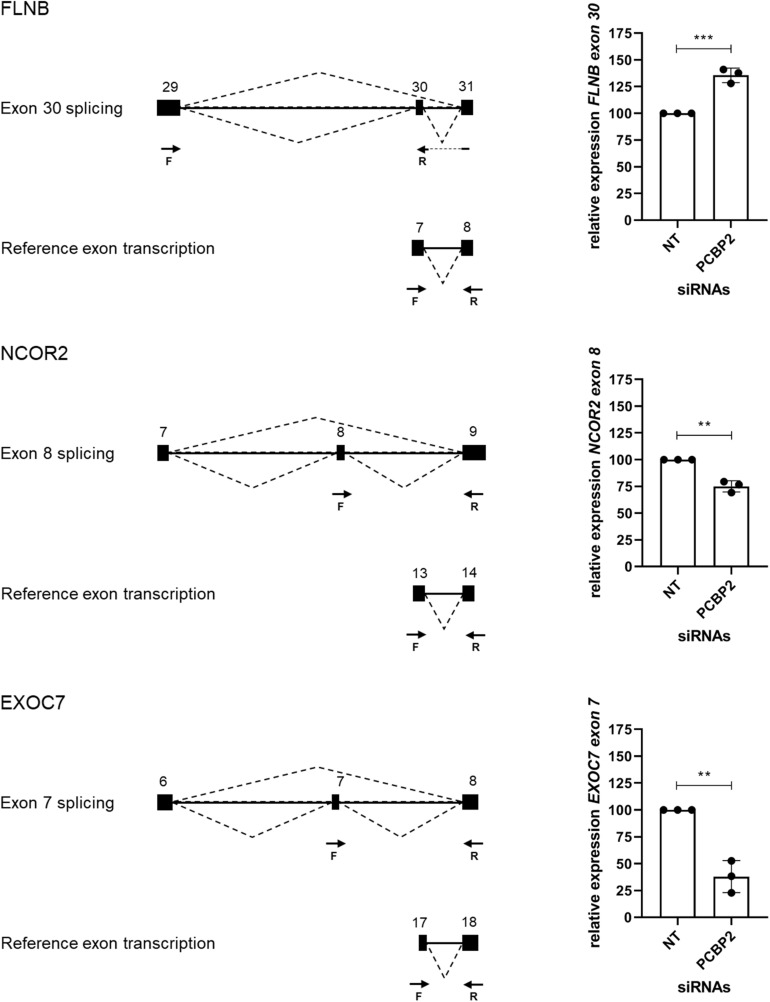
Validation of PCBP2 knockdown effect on splicing. Validation by qPCR of three genes identified by RNA-Seq to undergo transcript switching upon silencing of PCBP2. Transcription of reference exons of each of the genes was used for normalization. The specific included or excluded exons are depicted in the diagrams which also indicate the location of the primers used (forward [F] and reverse [R]). *FLNB* shows significant inclusion of exon 30 upon PCBP2 silencing, while *NCOR2* and *EXOC7* show significant exclusion of exon 8 and 7, respectively. Experiments were repeated three times using 3–4 replicates per treatment. Data are presented as mean ± SD and tested with a Student’s *t*-test. ** Indicates *p* < 0.01; *** indicates *p* < 0.001.

We validated the DRIMSeq results by two different approaches, i.e., quantitative PCR and a comparison with published results. To be able to validate the differential splicing effects by quantitative PCR we added four additional criteria: the transcript specific adjusted p value had to be < 0.05, the PCBP2/non-targeting ratio of the transcript proportion had to be > 1.5 or < 0.67, the gene had to be represented by at least two significant transcripts to be able to pinpoint the exon spliced differently between the two transcripts, and the mean counts of the transcript had to be at least 300 in the DRIMSeq analysis in either the non-targeting control or PCBP2 knockdown samples. The latter criterion was introduced to have enough copies to yield large enough differences to detect by qPCR based on preliminary qPCR experiments on detecting the published transcript switch occurring in *RUNX1* (Ghanem et al., 2018). This yielded a list of 10 genes, presented in Table 2. We chose three genes to validate by qPCR: two genes that showed exon exclusion upon PCBP2 knockdown, i.e., *NCOR2* and *EXOC7*, and one gene showing inclusion, i.e., *FLNB*. For each gene we designed primers recognizing the included/excluded exon and a set of primers recognizing all transcripts of the specific gene expressed in HTR-8/SVneo cells. We were able to validate differential splicing upon PCBP2 knockdown in all three genes (Figure 4).

For our second validation approach we used the data on differential splicing mediated by PCBP2 described in two publications (Ji et al., 2016; Ghanem et al., 2018) and summarized in Table 3. In these two publications transcript switching was studied upon co-silencing PCBP2 and PCBP1, except for studying *RUNX1* transcript usage in which specific knockdown of PCBP2 was performed. For *TARS2* and *CTTN* transcript expression analysis both co-silencing as well as silencing PCBP2 only was studied. We were able to confirm both genes that were already described to be differentially spliced upon PCBP2 silencing, i.e., *RUNX1* and *CTTN*. Additionally, for two genes (*CDK2* and *WNK4*) we could confirm significant exon exclusion upon PCBP2 knockdown. It should be noted that the differentially spliced transcripts of *RUNX1* and *WNK4* are not present in Supplementary Table 3 as the relative abundance proportion of the transcripts was below the cutoff value of 0.1. Taken together, by two distinct methods we were able to validate the results obtained by DRIMSeq analysis of the RNA-Seq data.

### PCBP2-Specific C-Rich Motif Adjacent to Skipped Exons

**FIGURE 5:**
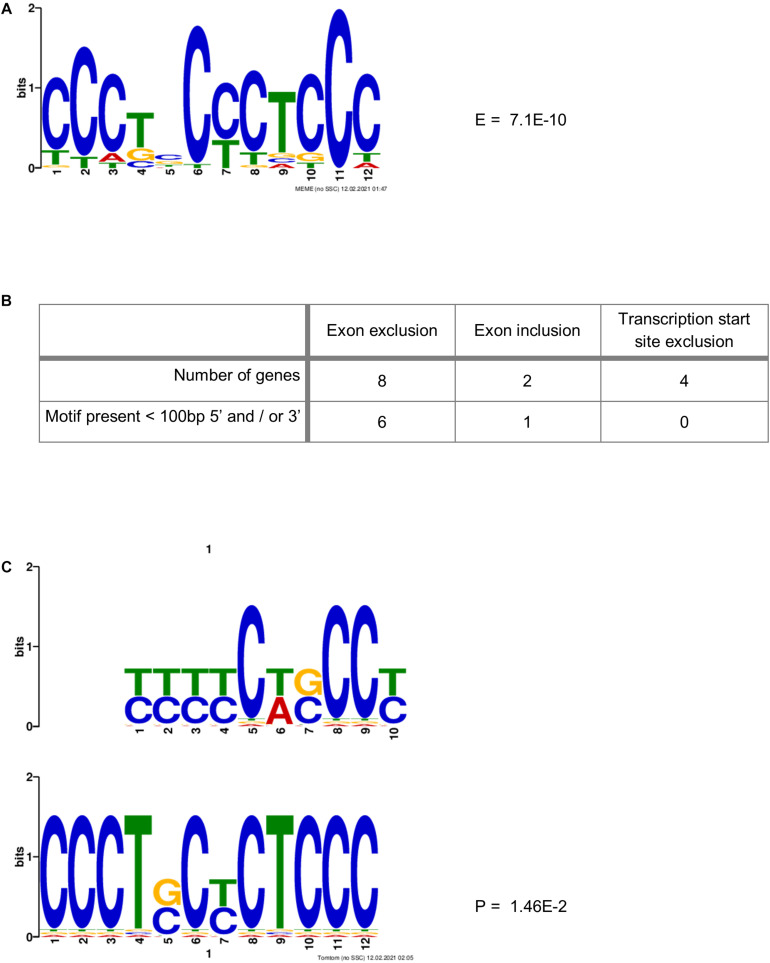
PCBP2-specific C-rich motif adjacent to exons skipped upon PCBP2 knockdown. **(A)** MEME motif enrichment analysis revealed a highly significant motif (E-value: 7.1E-10) in the intronic regions surrounding in- or excluded exons or transcription start sites upon PCBP2 silencing. **(B)** The motif was found in 75% of genes in regions within 100 bp from the exon boundaries of exons excluded upon PCBP2 silencing (see locations of motif in Supplementary Figure 2). The adjacent regions of excluded transcription start sites did not contain the motif, while the regions adjacent to the included exon of one of the two genes contained the motif. **(C)** The motif identified in the data set of this study (lower motif) was compared to the motif identified by Ji et al. (2016) (upper motif) by the Tomtom motif comparison tool which showed that the motifs significantly matched (*p*-value: 1.46E-2), but that they are not the same.

Ji et al. (2016) identified a motif (YYYYCWSCCY) that is highly enriched immediately adjacent (5′ and/or 3′) to the exons whose splicing was skipped upon co-depletion of PCBP1 and PCBP2. We therefore sought to identify a motif that might be more specific to PCBP2 only. This is because the data published by Ji et al. and the data presented in Table 3 suggest that both PCBP1 and PCBP2 can affect the splicing of certain exons but that this might be specific to either PCBP1 or PCBP2 in certain cases. We constructed a data file containing all intronic regions affected by PCBP2 knockdown (up to 500 bp) directly adjacent to the significantly excluded exons of the genes in Table 3 and added all intronic regions adjacent to all excluded exons of the genes of Table 2. To the data file we also added all intronic regions adjacent to included exons upon PCBP2 knockdown and added the 5′ and 3′ regions surrounding transcription start sites that were excluded upon PCBP2 knockdown. This data file was submitted to MEME motif enrichment analysis using a 0-order background model which provided us with a highly significant motif (CCCTSCYCTCCC), also depicted in Figure 5A. When studying the locations of the motif (Supplementary Figure 2) it was seen that in the group of genes that exclude an exon upon PCBP2 knockdown, the motif was found in 75% of the intronic regions < 100 bp 5′ and/or < 100 bp 3′ from the excluded exon (Figure 5B). The motif was additionally found in the adjacent regions in one of the two genes that included an exon upon PCBP2 silencing, but the motif was absent in the adjacent regions of the four genes with excluded transcription start sites upon PCBP2 knockdown. To be informed about the significance of the association between the presence of the motif adjacent to excluded exons compared to the presence of the motif adjacent to excluded transcription start sites and included exons a Fisher’s exact test was performed. Although proportionally apparent, the test did not reach statistical significance (*p* = 0.070) due to the low total number of regions included in the dataset. The motif identified in our small dataset was compared to the motif identified by Ji et al. (2016) using the Tomtom motif comparison tool (Bailey et al., 2015). This analysis indicated that the two motifs significantly match (Figure 5C), providing evidence that the motif we identified is highly comparable, but different, which might explain the higher specificity to PCBP2 induced splicing. Finally, we extracted data from the ENCODE project generated by Van Nostrand et al. (2020), which demonstrates direct binding of PCBP2 to several of the motif locations presented in Supplementary Figure 2. The ENCODE data were produced by performing enhanced CLIP (crosslinking and immunoprecipitation) *in vivo* binding experiments in HepG2 cells to identify binding of PCBP2 to its RNA targets. Within this dataset, although generated in a different cell type, PCBP2 is shown to directly bind to the actual motifs identified by our MEME analysis (Supplementary Figure 3), providing further evidence for the validity of the PCBP2-specific motif identified.

## Discussion

In this study we show by *in vitro* and *ex vivo* experiments that proteins directly bound to the HELLP syndrome associated long non-coding RNA *LINC-HELLP* are involved in the regulation of EVT invasion during placentation. In both HTR-8/SVneo EVT-like cells and in human first trimester placental explants we show that, upon knockdown, PCBP2, and to a lesser extend PCBP1 and YBX1, are able to negatively affect EVT differentiation through EMT inhibition. This was shown by a reduction in transwell invasion and an increase in proliferation in HTR-8/SVneo cells. First trimester explants treated with *PCBP2* siRNAs predominantly failed to produce any EVT outgrowth. Of the ones that still did show EVT outgrowth, the percentage of proliferating EVTs was significantly higher compared to controls.

We further investigated the effect of PCBP2 silencing on EVTs by performing whole transcriptome sequencing. Interestingly, PCBP2 knockdown only had a marginal effect on mRNA expression levels, providing evidence that the effect PCBP2 has on EVT differentiation does not occur through regulating downstream gene expression.

On the opposite, a large number of genes was found to show a switch in splicing upon PCBP2 silencing. Pathway analysis on the genes showing differential transcript usage revealed that these genes are associated with functions in cellular assembly and organization, cellular function and maintenance, and cellular growth and proliferation and the cell cycle. The majority of genes presented in Table 2 also conduct these functions. When EVTs differentiate from a proliferative epithelial cell to an invasive mesenchymal type of cell through Epithelial Mesenchymal Transition, they indeed alter their cellular assembly and their proliferative activity by exiting the cell cycle. Hereby they gain the capacity to invade the decidua of the mother.

Recently, Ruano et al. (2021) published the first systematic analysis of alternative splicing in third trimester placentas by microarray comparing preeclamptic and intra uterine growth restricted (IUGR) placentas to healthy controls. In their study, genes differentially spliced in preeclamptic placentas were predominantly associated with extracellular matrix organization pathways, genes differentially spliced in IUGR placentas were associated with hypoxia and hormone metabolism, while preeclampsia and IUGR both were associated with exocytosis. Especially the pathways associated with their third trimester preeclamptic placentas show overlap with the pathways identified in our PCBP2 silenced EVT-like cells. However, there are also some clear differences indicating that PCBP2, as can be expected, is not the sole regulator of alternative splicing occurring in placentas with pregnancy complications. The most interesting gene in this respect is *FLT1*, well known for its differential transcript usage in preeclampsia, where soluble transcripts of the gene are highly increased in preeclamptic pregnancies (Jebbink et al., 2011). Ruano et al. indeed show alternative splicing of *FLT1* in their third placental samples compared to controls, while *FLT1* is not differentially spliced in our dataset, indicating that PCBP2 does not regulate the splicing of *FLT1*.

For the majority of differentially spliced genes the actual effect on protein function is unknown and in pathway analysis it is merely assumed that the alternative transcript will lead to changes in function. However, for one of the genes validated by qPCR, *FLNB*, it actually has been proven that skipping of its exon 30 induces EMT as this exon encodes a hinge region that promotes EMT by releasing the FOXC1 transcription factor from an inhibitory complex (Li et al., 2018). In our study we confirm this result as we show that PCBP2 knockdown, reducing EMT, leads to inclusion of exon 30.

Additionally, in our study we identified a C-rich motif that is specifically located adjacent to exons that are excluded upon PCBP2 silencing. This motif is significantly comparable, but not the same, as the motif originally identified by Ji et al. (2016) who used co-depletion of PCBP1 and PCBP2. Our identified motif might therefore be more specific for binding and subsequent regulation by PCBP2.

To conclude, in this study we show that PCBP2, through mRNA splicing, might be a paramount regulator of extravillous trophoblast EMT differentiation, where its disturbed binding to *LINC-HELLP* could contribute to dysfunctional placental development as seen in the HELLP syndrome.

## Data Availability Statement

The datasets presented in this study can be found in online repositories. The names of the repository/repositories and accession number(s) can be found below: https://www.ncbi.nlm.nih.gov/, PRJNA691146.

## Ethics Statement

The studies involving human participants were reviewed and approved by Medical Ethical Committee of the Academic Medical Center, Amsterdam. The patients/participants provided their written informed consent to participate in this study.

## Author Contributions

GA and MD designed the study. DG, SB, and DJ performed the experiments. DG, SB, DJ, and MD analyzed the data. RK and GA performed the RNA-Seq analysis. DG and MD wrote the manuscript. All authors reviewed and approved the final version of the manuscript.

## Conflict of Interest

The authors declare that the research was conducted in the absence of any commercial or financial relationships that could be construed as a potential conflict of interest.
