# Supplementary material for: Knockdown of Splicing Complex Protein PCBP2 Reduces Extravillous Trophoblast Differentiation Through Transcript Switching
Source: Front Cell Dev Biol. 2021 May 20;9:671806. doi: 10.3389/fcell.2021.671806 (PMC8172583; doi:10.3389/fcell.2021.671806)
Supplement: Supplementary file 1 [file Data_Sheet_1.PDF]

Supplementary Material

Knockdown of splicing complex protein PCBP2 reduces extravillous trophoblast differentiation through transcript switching

Danai Georgiadou, Souad Boussata, Remco Keijser, Dianta AM Janssen, Gijs B Afink, Marie van Dijk

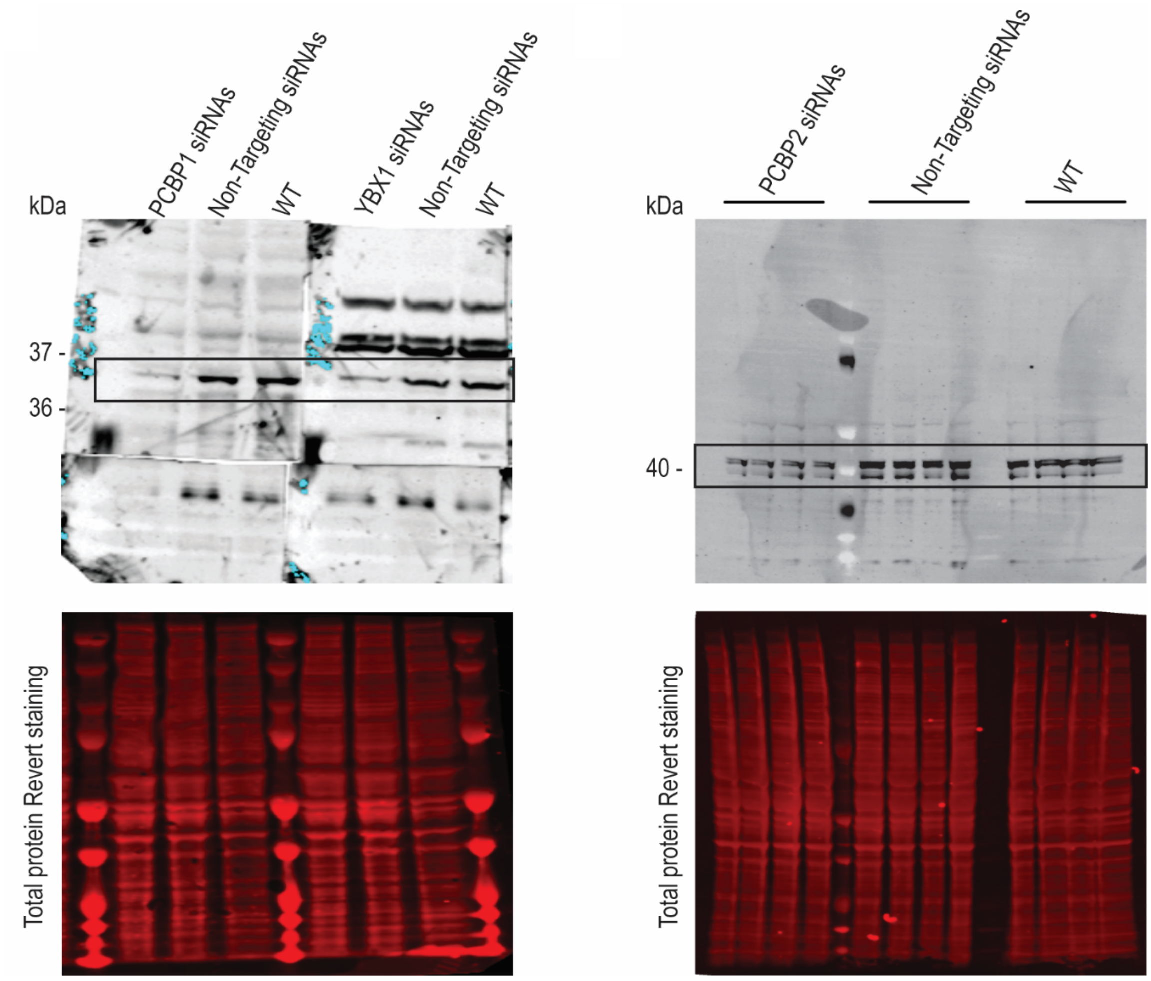

Supplementary Figure 1. Total protein Revert stain (Red) and uncropped pictures of the blots (Black & White) presented in Figure 1B.

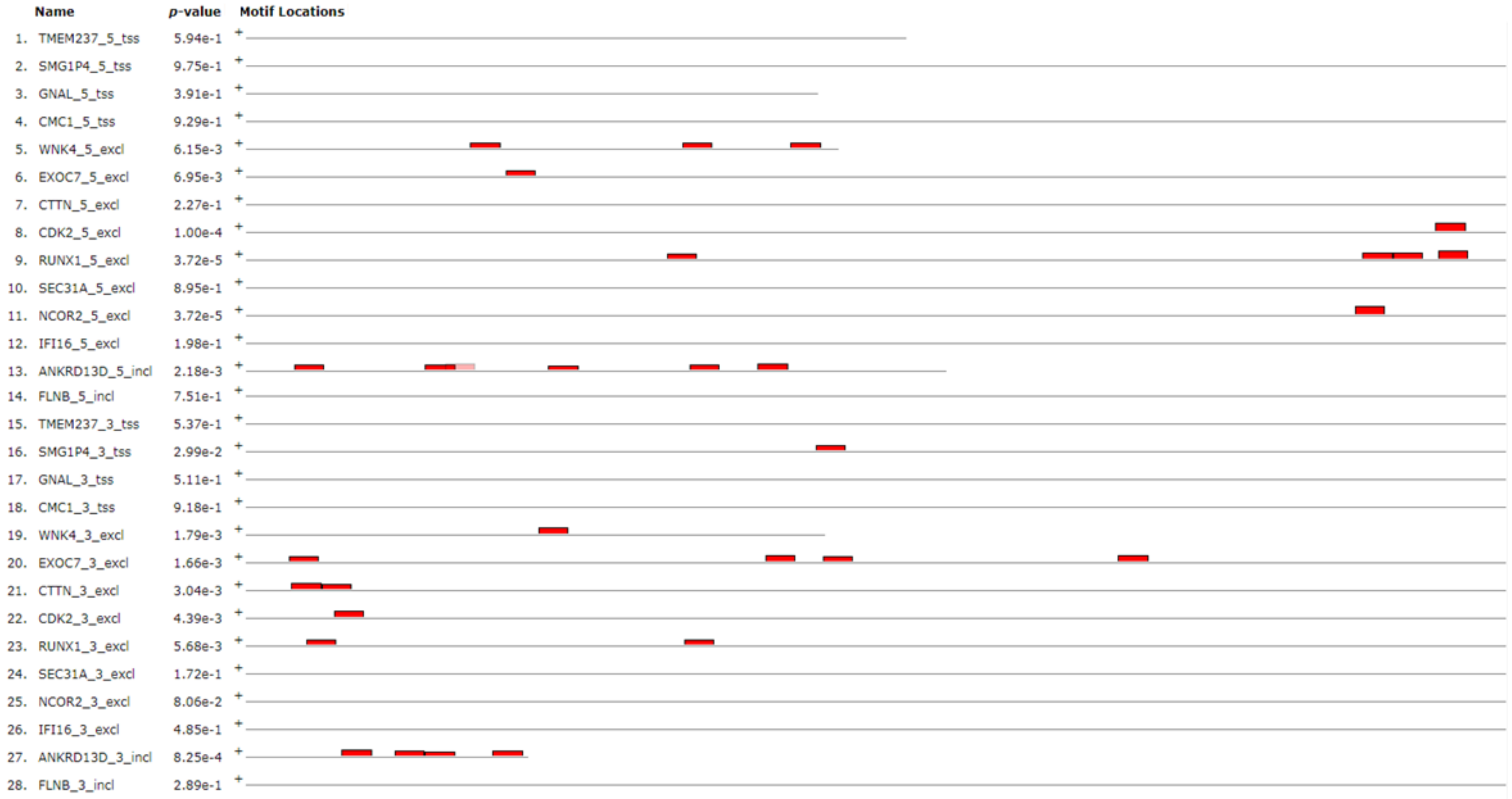

Supplementary Figure 2. Locations of the identified motif (red boxes) in intronic regions 5' and 3' of excluded exons (excl), included exons (incl) or excluded transcription start sites (tss). The lines represent the 500 bp intronic region, or less when the actual intron was smaller, adjacent to the included or excluded exon or transcription start site. The +-sign indicates that the motifs were identified on the positive strand only since all regions were introduced into the MEME analysis using the coding strand of the respective genes.

Supplementary Figure 3. On the following pages data extracted from the ENCODE project is shown providing evidence of direct binding of PCBP2 to several of the motif locations shown in Supplementary Figure 2. The ENCODE data were produced by performing enhanced CLIP (crosslinking and immunoprecipitation) in vivo binding experiments in HepG2 cells to identify binding of PCBP2 to its RNA targets. Two replicate experiments were done (PCBP2 rep1 and PCBP2 rep2) and controlled using a size-matched input control experiment (Unknown Target rep1). Peak calling by the eCLIP analysis pipeline resulted in significantly enriched peaks as shown in the upper two tracks of the UCSC Genome Browser plots below. The actual motifs, as identified by the MEME analysis, within these significantly enriched peaks are boxed in red.

EXOC7

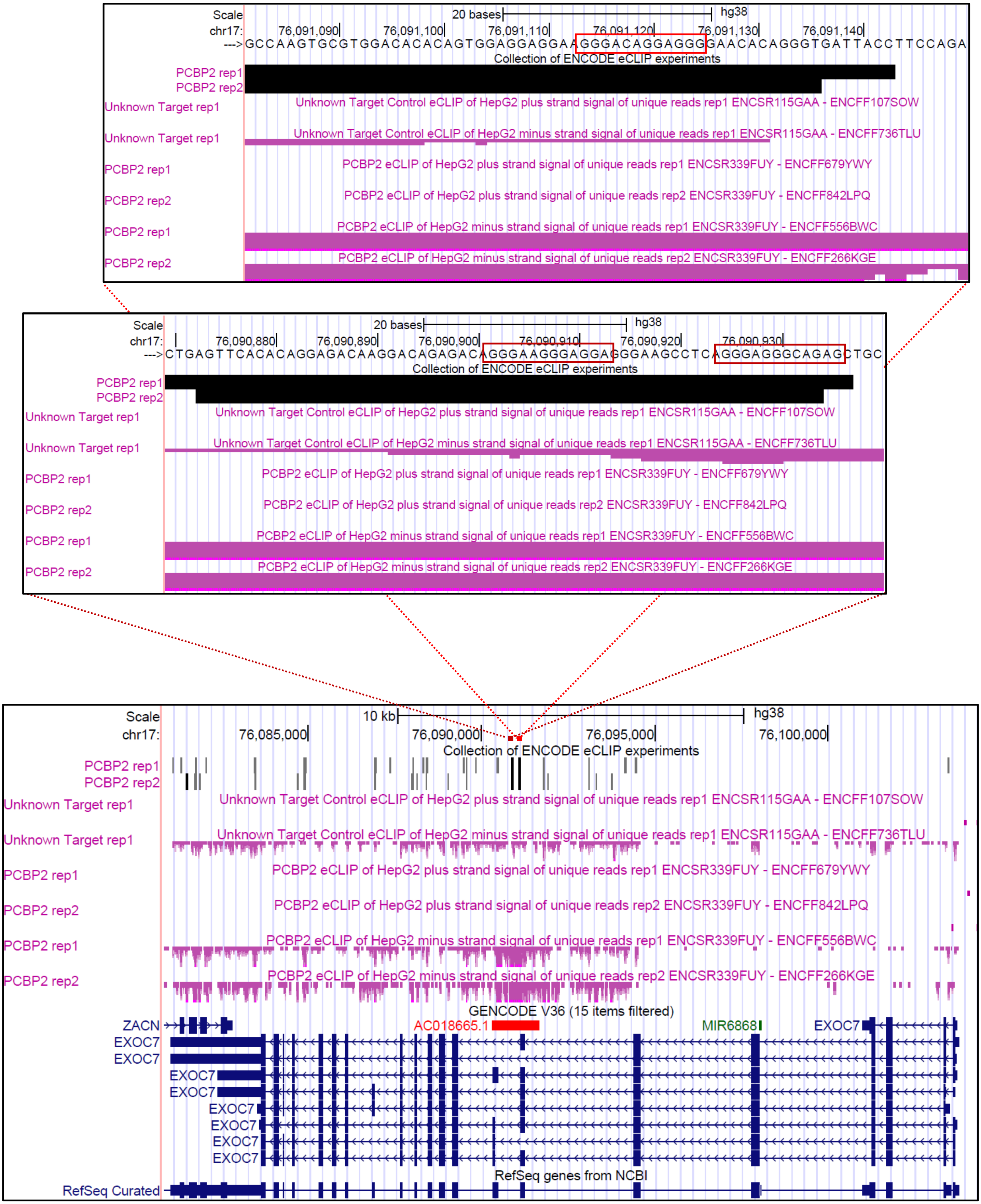

Scale
20 bases
hg38
chr17:
76,091,090
76,091,100
76,091,110
76,091,120
76,091,130
76,091,140
--->GCCAAGTGCGTGGACACACAGTGGAGGAGGAAGGACAGGAGGGGAACACAGGGTGATTACCTTCCAGA
Collection of ENCODE eCLIP experiments
PCBP2 rep1
PCBP2 rep2
Unknown Target rep1
Unknown Target Control eCLIP of HepG2 plus strand signal of unique reads rep1 ENCSR115GAA - ENCFF107SOW
Unknown Target rep1
Unknown Target Control eCLIP of HepG2 minus strand signal of unique reads rep1 ENCSR115GAA - ENCFF736TLU
PCBP2 rep1
PCBP2 eCLIP of HepG2 plus strand signal of unique reads rep1 ENCSR339FUY - ENCFF679YWY
PCBP2 rep2
PCBP2 eCLIP of HepG2 plus strand signal of unique reads rep2 ENCSR339FUY - ENCFF842LPQ
PCBP2 rep1
PCBP2 eCLIP of HepG2 minus strand signal of unique reads rep1 ENCSR339FUY - ENCFF556BWC
PCBP2 rep2
PCBP2 eCLIP of HepG2 minus strand signal of unique reads rep2 ENCSR339FUY - ENCFF266KGE
Scale
20 bases
hg38
chr17:
76,090,880
76,090,890
76,090,900
76,090,910
76,090,920
76,090,930
--->CTGAGTTCACACAGGAGACAAGGACAGAGACAGGGAAGGGAGGAGGGAAGCCTCAGGGAGGGCAGAGCTGC
Collection of ENCODE eCLIP experiments
Scale
10 kb
hg38
chr17:
76,085,000
76,090,000
76,095,000
76,100,000
GENCODE V36 (15 items filtered)
ZACN
AC018665.1
MIR6868
EXOC7
RefSeq genes from NCBI
RefSeq Curated

CTTN

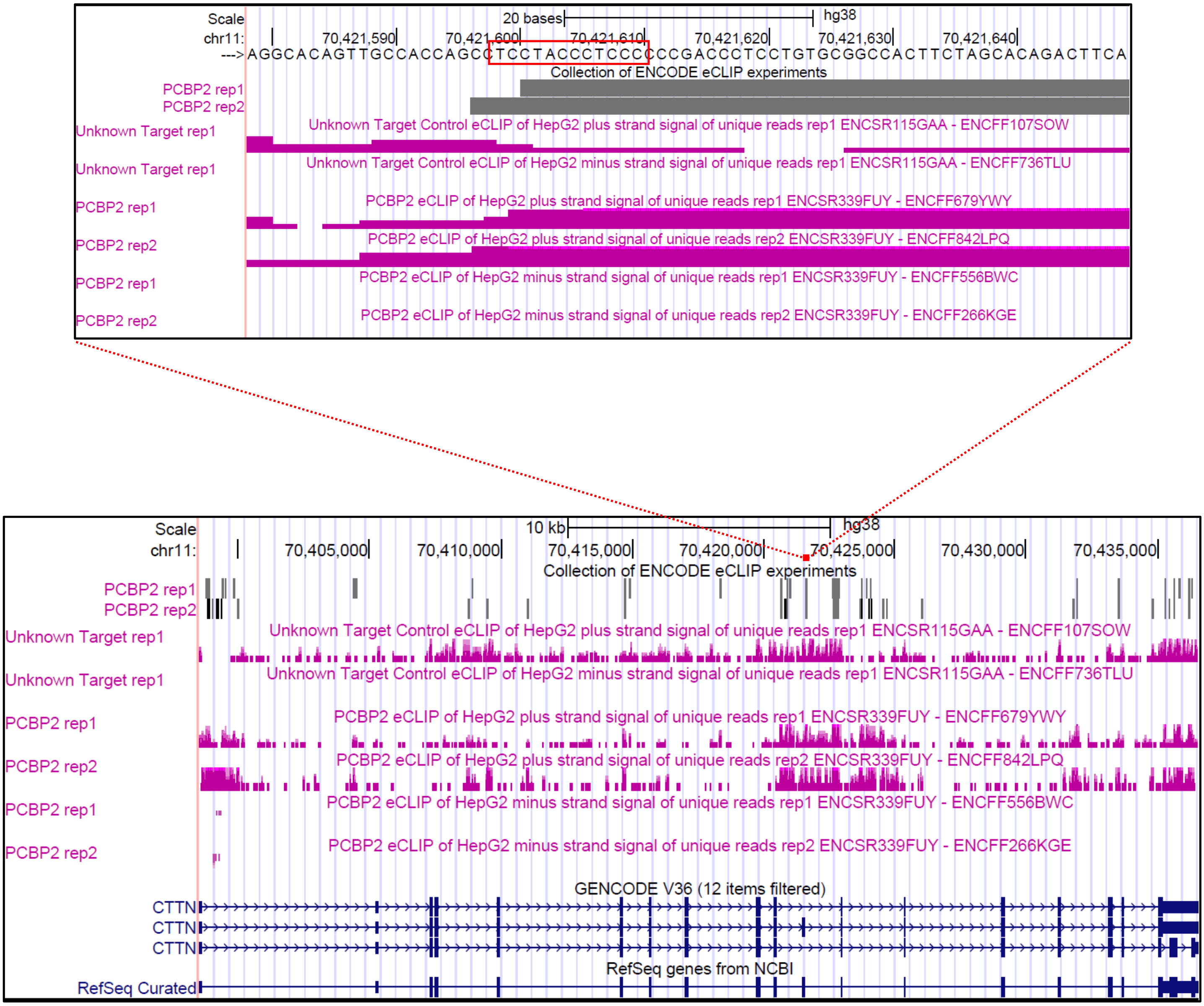
Scale
chr11:
20 bases
hg38
70,421,590
70,421,600
70,421,610
70,421,620
70,421,630
70,421,640
---> AGGCACAGTTGCCACCAGCCTCCTACCCTCCCCCCGACCCTCCTGTGCGGCCACTTCTAGCACAGACTTCA
Collection of ENCODE eCLIP experiments
PCBP2 rep1
PCBP2 rep2
Unknown Target rep1
Unknown Target Control eCLIP of HepG2 plus strand signal of unique reads rep1 ENCSR115GAA - ENCFF107SOW
Unknown Target rep1
Unknown Target Control eCLIP of HepG2 minus strand signal of unique reads rep1 ENCSR115GAA - ENCFF736TLU
PCBP2 rep1
PCBP2 eCLIP of HepG2 plus strand signal of unique reads rep1 ENCSR339FUY - ENCFF679YWY
PCBP2 rep2
PCBP2 eCLIP of HepG2 plus strand signal of unique reads rep2 ENCSR339FUY - ENCFF842LPQ
PCBP2 rep1
PCBP2 eCLIP of HepG2 minus strand signal of unique reads rep1 ENCSR339FUY - ENCFF556BWC
PCBP2 rep2
PCBP2 eCLIP of HepG2 minus strand signal of unique reads rep2 ENCSR339FUY - ENCFF266KGE
Scale
chr11:
10 kb
hg38
70,405,000
70,410,000
70,415,000
70,420,000
70,425,000
70,430,000
70,435,000
Collection of ENCODE eCLIP experiments
PCBP2 rep1
PCBP2 rep2
Unknown Target rep1
Unknown Target Control eCLIP of HepG2 plus strand signal of unique reads rep1 ENCSR115GAA - ENCFF107SOW
Unknown Target rep1
Unknown Target Control eCLIP of HepG2 minus strand signal of unique reads rep1 ENCSR115GAA - ENCFF736TLU
PCBP2 rep1
PCBP2 eCLIP of HepG2 plus strand signal of unique reads rep1 ENCSR339FUY - ENCFF679YWY
PCBP2 rep2
PCBP2 eCLIP of HepG2 plus strand signal of unique reads rep2 ENCSR339FUY - ENCFF842LPQ
PCBP2 rep1
PCBP2 eCLIP of HepG2 minus strand signal of unique reads rep1 ENCSR339FUY - ENCFF556BWC
PCBP2 rep2
PCBP2 eCLIP of HepG2 minus strand signal of unique reads rep2 ENCSR339FUY - ENCFF266KGE
GENCODE V36 (12 items filtered)
CTTN
CTTN
CTTN
RefSeq genes from NCBI
RefSeq Curated

WNK4

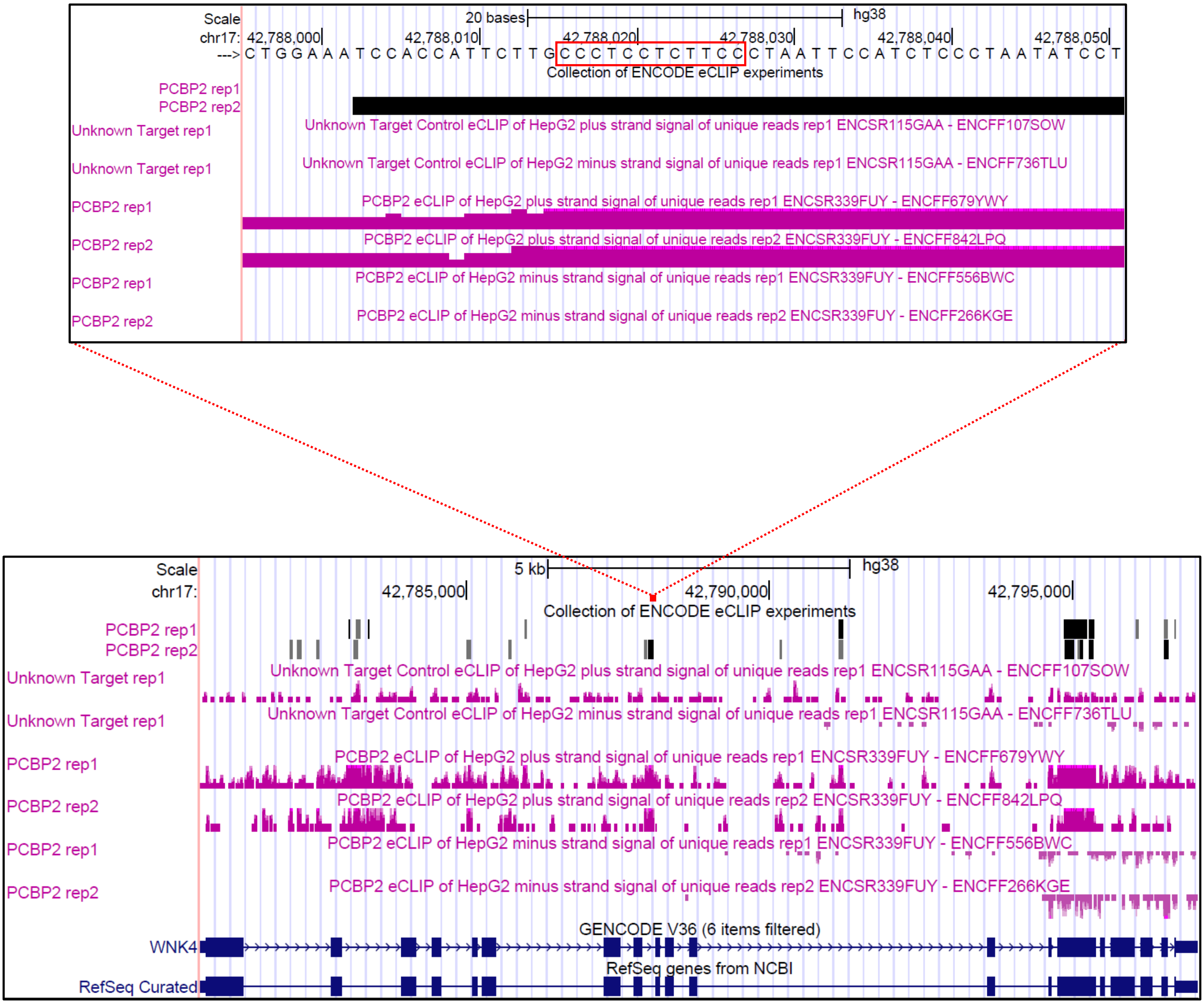
Scale
20 bases
hg38
chr17:
42,788,000
42,788,010
42,788,020
42,788,030
42,788,040
42,788,050
---> C T G G A A A T C C A C C A T T C T T G C C C T C C T C T T C C C T A A T T C C A T C T C C C T A A T A T C C T
Collection of ENCODE eCLIP experiments
PCBP2 rep1
PCBP2 rep2
Unknown Target rep1
Unknown Target Control eCLIP of HepG2 plus strand signal of unique reads rep1 ENCSR115GAA - ENCFF107SOW
Unknown Target rep1
Unknown Target Control eCLIP of HepG2 minus strand signal of unique reads rep1 ENCSR115GAA - ENCFF736TLU
PCBP2 rep1
PCBP2 eCLIP of HepG2 plus strand signal of unique reads rep1 ENCSR339FUY - ENCFF679YWY
PCBP2 rep2
PCBP2 eCLIP of HepG2 plus strand signal of unique reads rep2 ENCSR339FUY - ENCFF842LPQ
PCBP2 rep1
PCBP2 eCLIP of HepG2 minus strand signal of unique reads rep1 ENCSR339FUY - ENCFF556BWC
PCBP2 rep2
PCBP2 eCLIP of HepG2 minus strand signal of unique reads rep2 ENCSR339FUY - ENCFF266KGE
Scale
5 kb
hg38
chr17:
42,785,000
42,790,000
42,795,000
Collection of ENCODE eCLIP experiments
PCBP2 rep1
PCBP2 rep2
Unknown Target rep1
Unknown Target Control eCLIP of HepG2 plus strand signal of unique reads rep1 ENCSR115GAA - ENCFF107SOW
Unknown Target rep1
Unknown Target Control eCLIP of HepG2 minus strand signal of unique reads rep1 ENCSR115GAA - ENCFF736TLU
PCBP2 rep1
PCBP2 eCLIP of HepG2 plus strand signal of unique reads rep1 ENCSR339FUY - ENCFF679YWY
PCBP2 rep2
PCBP2 eCLIP of HepG2 plus strand signal of unique reads rep2 ENCSR339FUY - ENCFF842LPQ
PCBP2 rep1
PCBP2 eCLIP of HepG2 minus strand signal of unique reads rep1 ENCSR339FUY - ENCFF556BWC
PCBP2 rep2
PCBP2 eCLIP of HepG2 minus strand signal of unique reads rep2 ENCSR339FUY - ENCFF266KGE
GENCODE V36 (6 items filtered)
WNK4
RefSeq genes from NCBI
RefSeq Curated

RUNX1

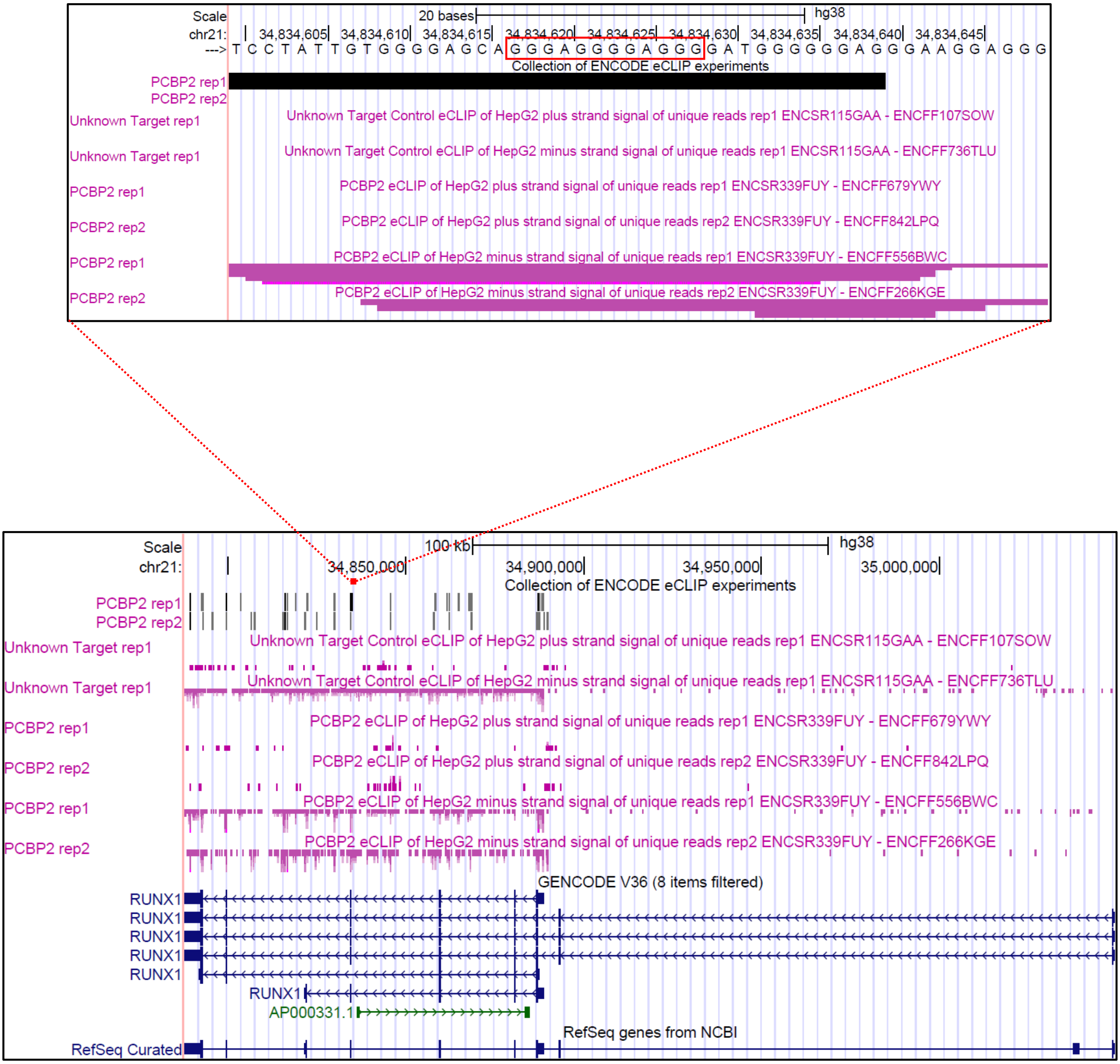

Scale
20 bases
hg38
chr21:
34,834,605
34,834,610
34,834,615
34,834,620
34,834,625
34,834,630
34,834,635
34,834,640
34,834,645
---> T C C T A T T G T G G G G A G C A G G G A G G G A G G G A T G G G G G G A G G G A A G G A G G G
Collection of ENCODE eCLIP experiments
PCBP2 rep1
PCBP2 rep2
Unknown Target rep1
Unknown Target Control eCLIP of HepG2 plus strand signal of unique reads rep1 ENCSR115GAA - ENCFF107SOW
Unknown Target rep1
Unknown Target Control eCLIP of HepG2 minus strand signal of unique reads rep1 ENCSR115GAA - ENCFF736TLU
PCBP2 rep1
PCBP2 eCLIP of HepG2 plus strand signal of unique reads rep1 ENCSR339FUY - ENCFF679YWY
PCBP2 rep2
PCBP2 eCLIP of HepG2 plus strand signal of unique reads rep2 ENCSR339FUY - ENCFF842LPQ
PCBP2 rep1
PCBP2 eCLIP of HepG2 minus strand signal of unique reads rep1 ENCSR339FUY - ENCFF556BWC
PCBP2 rep2
PCBP2 eCLIP of HepG2 minus strand signal of unique reads rep2 ENCSR339FUY - ENCFF266KGE
Scale
100 kb
hg38
chr21:
34,850,000
34,900,000
34,950,000
35,000,000
Collection of ENCODE eCLIP experiments
PCBP2 rep1
PCBP2 rep2
Unknown Target rep1
Unknown Target Control eCLIP of HepG2 plus strand signal of unique reads rep1 ENCSR115GAA - ENCFF107SOW
Unknown Target rep1
Unknown Target Control eCLIP of HepG2 minus strand signal of unique reads rep1 ENCSR115GAA - ENCFF736TLU
PCBP2 rep1
PCBP2 eCLIP of HepG2 plus strand signal of unique reads rep1 ENCSR339FUY - ENCFF679YWY
PCBP2 rep2
PCBP2 eCLIP of HepG2 plus strand signal of unique reads rep2 ENCSR339FUY - ENCFF842LPQ
PCBP2 rep1
PCBP2 eCLIP of HepG2 minus strand signal of unique reads rep1 ENCSR339FUY - ENCFF556BWC
PCBP2 rep2
PCBP2 eCLIP of HepG2 minus strand signal of unique reads rep2 ENCSR339FUY - ENCFF266KGE
GENCODE V36 (8 items filtered)
RUNX1
RUNX1
RUNX1
RUNX1
RUNX1
RUNX1
AP000331.1
RefSeq genes from NCBI
RefSeq Curated

ANKRD13D

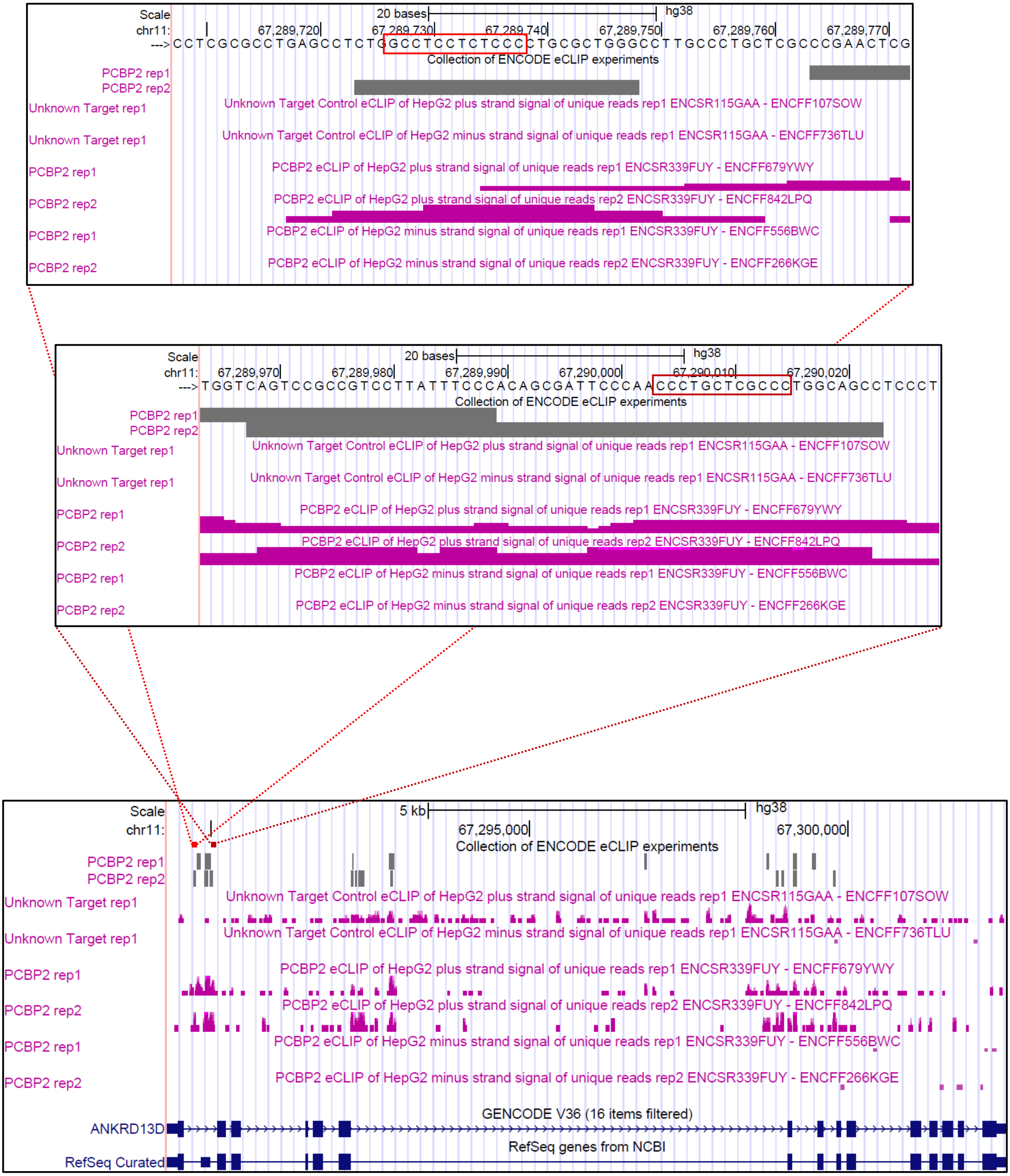

Scale
20 bases
hg38
chr11:
67,289,720
67,289,730
67,289,740
67,289,750
67,289,760
67,289,770
---> CCTCGCGCCTGAGCCTCTGGCCTCCTCTCCCCTGCGCTGGGCCTTGCCCTGCTCGCCCGAACTCG
Collection of ENCODE eCLIP experiments
PCBP2 rep1
PCBP2 rep2
Unknown Target rep1
Unknown Target Control eCLIP of HepG2 plus strand signal of unique reads rep1 ENCSR115GAA - ENCFF107SOW
Unknown Target rep1
Unknown Target Control eCLIP of HepG2 minus strand signal of unique reads rep1 ENCSR115GAA - ENCFF736TLU
PCBP2 rep1
PCBP2 eCLIP of HepG2 plus strand signal of unique reads rep1 ENCSR339FUY - ENCFF679YWY
PCBP2 rep2
PCBP2 eCLIP of HepG2 plus strand signal of unique reads rep2 ENCSR339FUY - ENCFF842LPQ
PCBP2 rep1
PCBP2 eCLIP of HepG2 minus strand signal of unique reads rep1 ENCSR339FUY - ENCFF556BWC
PCBP2 rep2
PCBP2 eCLIP of HepG2 minus strand signal of unique reads rep2 ENCSR339FUY - ENCFF266KGE
Scale
20 bases
hg38
chr11:
67,289,970
67,289,980
67,289,990
67,290,000
67,290,010
67,290,020
---> TGGTCAGTCCGCCGTCCTTATTTCCCACAGCGATTCCCAACCCTGCTCGCCCTGGCAGCCTCCCT
Collection of ENCODE eCLIP experiments
PCBP2 rep1
PCBP2 rep2
Unknown Target rep1
Unknown Target Control eCLIP of HepG2 plus strand signal of unique reads rep1 ENCSR115GAA - ENCFF107SOW
Unknown Target rep1
Unknown Target Control eCLIP of HepG2 minus strand signal of unique reads rep1 ENCSR115GAA - ENCFF736TLU
PCBP2 rep1
PCBP2 eCLIP of HepG2 plus strand signal of unique reads rep1 ENCSR339FUY - ENCFF679YWY
PCBP2 rep2
PCBP2 eCLIP of HepG2 plus strand signal of unique reads rep2 ENCSR339FUY - ENCFF842LPQ
PCBP2 rep1
PCBP2 eCLIP of HepG2 minus strand signal of unique reads rep1 ENCSR339FUY - ENCFF556BWC
PCBP2 rep2
PCBP2 eCLIP of HepG2 minus strand signal of unique reads rep2 ENCSR339FUY - ENCFF266KGE
Scale
5 kb
hg38
chr11:
67,295,000
67,300,000
Collection of ENCODE eCLIP experiments
PCBP2 rep1
PCBP2 rep2
Unknown Target rep1
Unknown Target Control eCLIP of HepG2 plus strand signal of unique reads rep1 ENCSR115GAA - ENCFF107SOW
Unknown Target rep1
Unknown Target Control eCLIP of HepG2 minus strand signal of unique reads rep1 ENCSR115GAA - ENCFF736TLU
PCBP2 rep1
PCBP2 eCLIP of HepG2 plus strand signal of unique reads rep1 ENCSR339FUY - ENCFF679YWY
PCBP2 rep2
PCBP2 eCLIP of HepG2 plus strand signal of unique reads rep2 ENCSR339FUY - ENCFF842LPQ
PCBP2 rep1
PCBP2 eCLIP of HepG2 minus strand signal of unique reads rep1 ENCSR339FUY - ENCFF556BWC
PCBP2 rep2
PCBP2 eCLIP of HepG2 minus strand signal of unique reads rep2 ENCSR339FUY - ENCFF266KGE
GENCODE V36 (16 items filtered)
ANKRD13D
RefSeq genes from NCBI
RefSeq Curated

Gene	Forward (5’- 3’)	Reverse (5’- 3’)
YBX1	CAACAGGAATGACACCAAGGAA	TCCTACACTGCGAAGGTACT
PCBP1	ACTTGACCACGTAACGAGCC	ACTTTCAGTCACACCGGCAT
PCBP2	TAGCATATCCCCTGCCACCT	TGCTAGTCAGTGTGGCTCTC
PSMD4	GGCAAGATCACCTTCTGCAC	CTTCCCACAAAGGCAATGAT
YWHAZ	AATGGCTTCATCGAAAGCTG	CTGGCCCTCAACTTCTCTGT
FLNB exon 30	CCTTCACTGTCATGGCCACA	CTCTTCGGTCACCCAGGG
FLNB ref. exons	CAGGGTTGGAAGCTGTAGGG	TCTTCCACGAGCAACTCCAC
NCOR2 exon 8	GGAATGAGGTGCCCAGAGG	ATTCTGCCCTGTGTCCTTGG
NCOR2 ref. exons	CCTCATCTCCTCTGCCAAGC	GCCTTGGCATGCTCTGAGTA
EXOC7 exon 7	GGGGCTCTAACCTCATTCCTC	TCTTCTTCTGGTGGTGCTCG
EXOC7 ref. exons	GGACAAGGAGCGGCAGATTA	ACAATGGTCTTCTGGGCCTG

Supplementary Table 1. qPCR primer sequences.
